# Cell cycle progression data on human skin cancer cells with anticancer synthetic peptide LTX-315 treatment

**DOI:** 10.1016/j.dib.2020.105443

**Published:** 2020-03-19

**Authors:** Gloria A. Santa-González, Edwin Patiño-González, Marcela Manrique-Moreno

**Affiliations:** a Genetic Regeneration and Cancer Group, Faculty of Exact and Natural Sciences, Biology Institute, University of Antioquia, A.A. 1226, Medellin, Colombia; b Structural Biochemistry of Macromolecules Group, Faculty of Exact and Natural Sciences, Chemistry Institute, University of Antioquia, A.A. 1226, Medellin, Colombia

**Keywords:** Skin cancer, Therapeutic peptides, Cell cycle, Apoptosis, LTX-315

## Abstract

Skin cancer, including melanoma and non-melanoma (NMSC), represents the most common type of malignancy in the white population [1]. The incidence rate of melanoma is increasing worldwide, while the associated mortality remains stable. On the other hand, the incidence of NMSC varies widely [1,2]. Camilio and collaborators recently described the anticancer properties of LTX-315, a novel synthetic anticancer peptide, commercialized as Oncopore™ [3,4]. Despite various studies demonstrating the efficiency of LTX-315 therapy in inducing cancer cell death, the effects on cell cycle progression of this antitumoral peptide are poorly understood. In this research, we present data about the effect of LTX-315 on the cell cycle of two skin cancer cell lines: epidermoid carcinoma cells (A431) and melanoma cells (A375); as well as on an immortalized normal keratinocyte cell line, HaCaT. Additionally, its cytotoxicity on the cells was determined by measuring the uptake of propidium iodide, in order to establish its relationship with cell cycle progression. The analysed data obtained by flow cytometry show different cell cycle distributions in non-tumoral and skin cancer-derived cell lines in response to LTX-315 treatment. Non-tumoral cells showed a sub-G1 peak, while for tumoral cells there was a shift in the G1peak without producing an obvious distant and distinct sub-G1 peak. This data is in accordance with a major decrease in cell viability in non-cancer cells.

Specifications table

**Table:** 

Subject	Biochemistry, Genetics and molecular biology
Specific subject area	Cancer research cell biology
Type of data	Raw, graph and figure
How data were acquired	Flow cytometry (BD LSRFortessa)
Data format	Analyzed data with FlowJo software and raw data (.fcs files)
Parameters for data collection	Percentage of cell cycle phase distributions and cell viability of cancer cells treated with LTX-315 in counterpart with non-treated cells and non-tumoral cells.
Description of data collection	Flow cytometry analyses were performed to quantified DNA content and propidium iodide uptake, in order to determine cell cycle progression and membrane permeability.
Data source location	Medellin, Colombia
Data accessibility	The raw data files are provided in the data in brief dataverse https://doi.org/10.7910/DVN/LO4URR and https://doi.org/10.7910/DVN/ILRIKU All other data is with this article

## Value of the data

• The data provide the first experimental evidence about the effect of the anticancer peptide LTX-315 on the distribution of cell cycle phases in normal and skin cancer cells.
• Dataset allows comparison between LTX-315 concentrations and cell cycle distributions in two different skin cancer cell lines and in non-tumoral cells.
• The data provide valuable information on the relationships between cell cycle distribution and cell viability in non-tumoral and skin cancer-derived cells in response to LTX-315 treatment.
• Researchers can use the data as additional evidence to investigate the selectivity of the treatment with LTX-315.

## 2 Data

**Fig. 1:**
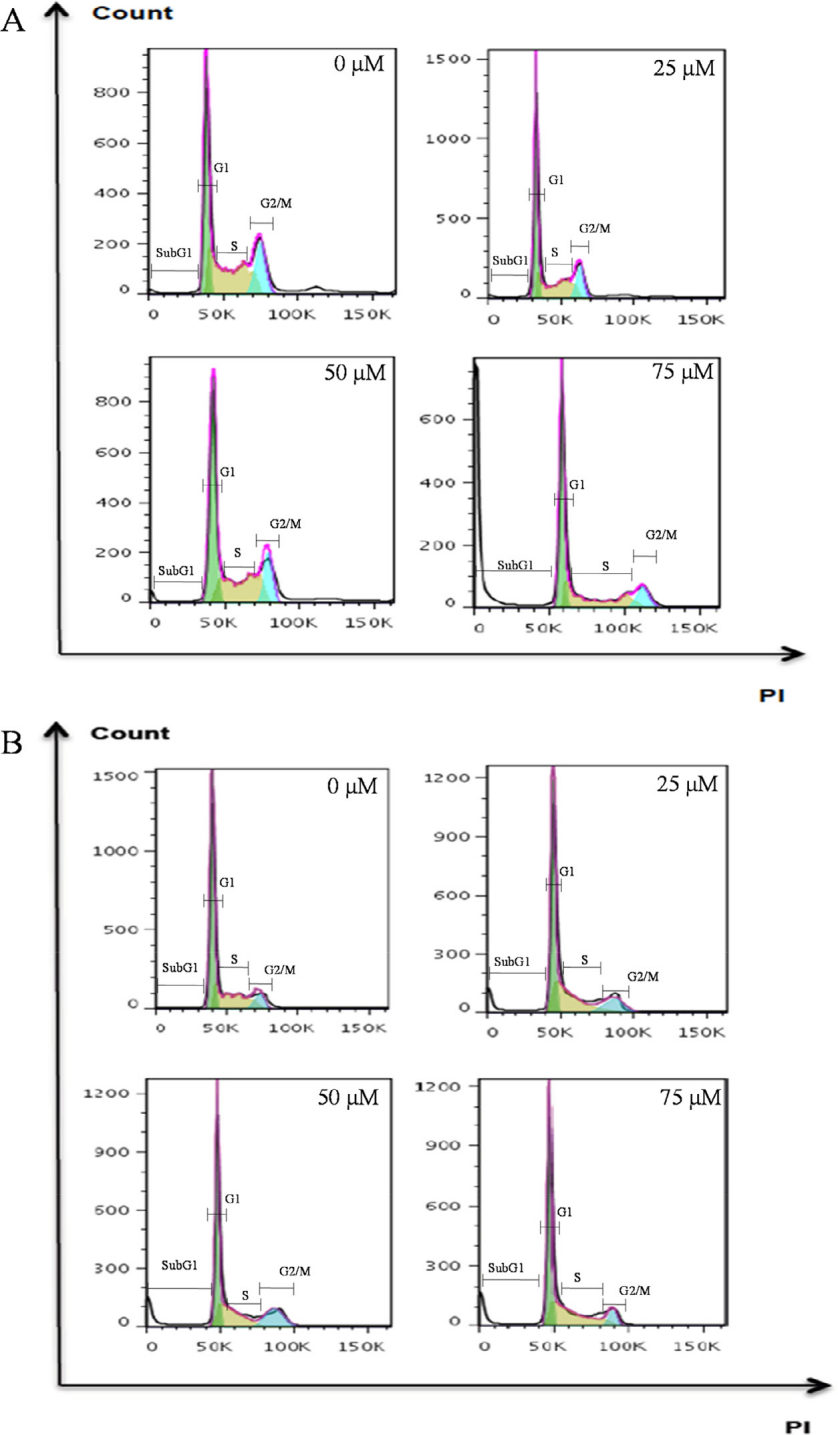

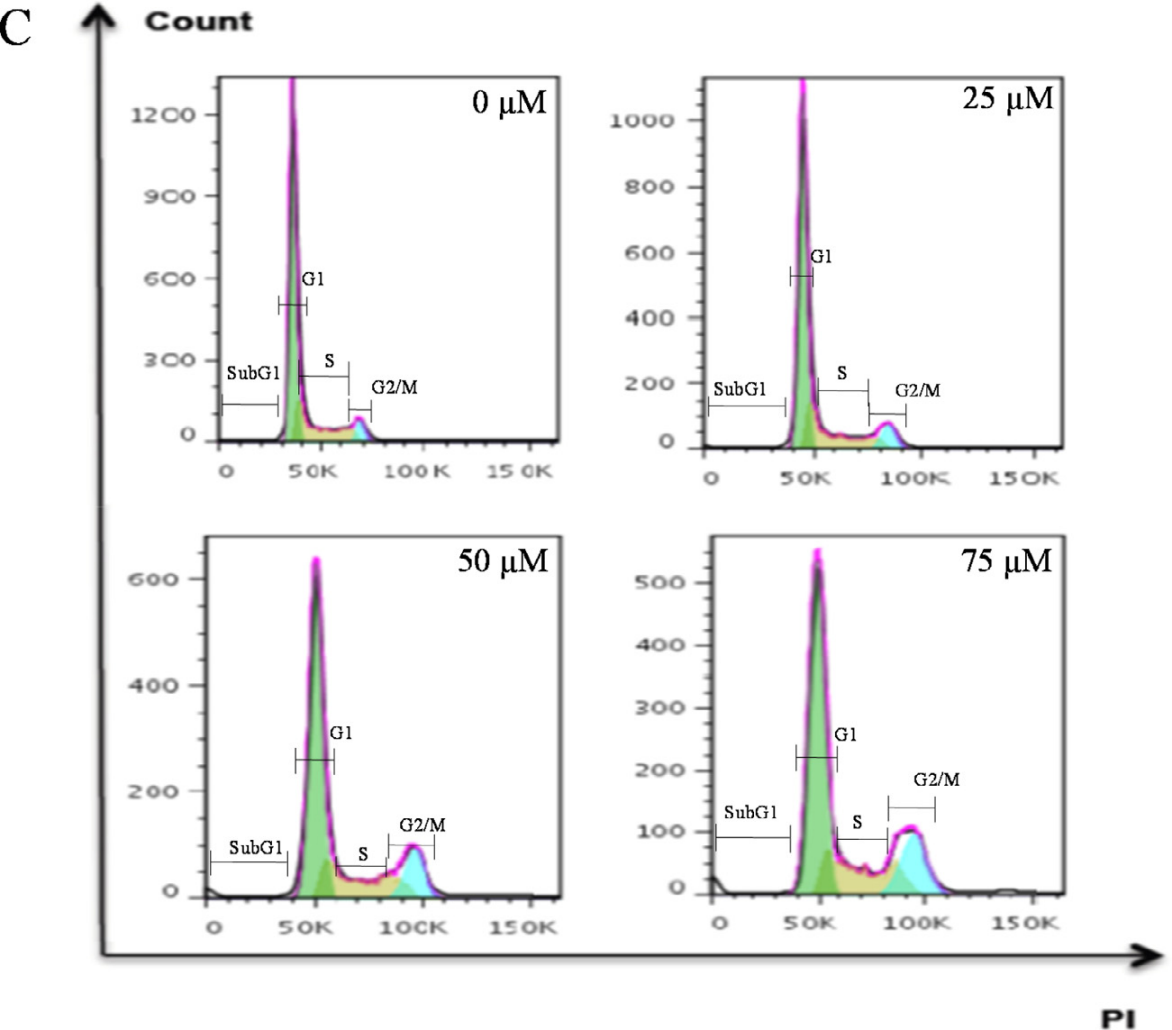
Cell cycle distribution in non-tumoral and cancer cells after LTX-315 treatment. Cells were treated with different concentrations of LTX-315 for 24 h. A. HaCaT, normal human keratinocyte cell line. B. A431, epidermoid carcinoma cells C. A375, melanoma cancer cells. The histograms show a representative example of the distribution of cell cycle phases in each treatment.

**Fig. 2:**
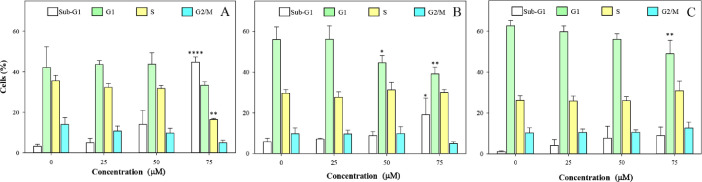
Quantification of cell cycle distribution of the total cell population in the different phases of the cell cycle. A. HaCaT, normal human keratinocyte cell line. B. A431, epidermoid carcinoma cells C. A375, melanoma cancer cells. Values are expressed as mean ± SEM of three independent experiments. Two-way ANOVA for sub-G1, G1, S and G2/M populations, difference with respect to NT, * *p* ≤ 0.05, ** *p* ≤ 0.01, **** *p* ≤ 0.0001.

**Fig. 3:**
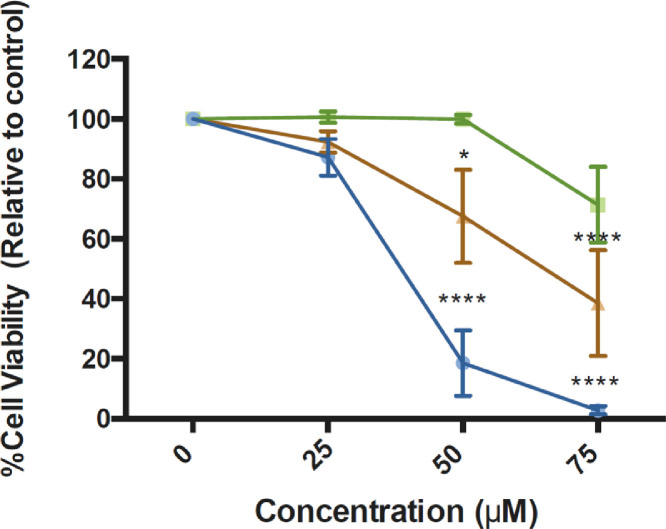
Cell viability percentages after treatment with LTX-315 in non-tumoral HaCaT (), cancer cell lines A431 () and A375 (). Cells were treated with different concentrations of LTX-315 for 24 h, after which propidium iodide uptake was assayed, as a measure of membrane integrity. Non-treated cells were considered as having 100% viability. Values are expressed as mean ± SEM of three independent experiments. Two-way ANOVA, difference with respect to NT, * *p* ≤ 0.05, **** *p* ≤ 0.0001.

Histograms (Fig. 1) and bar graphs (Fig. 2) showed that treatment with peptide LTX-315, compared to non-treated cells, induced a sub-G1 peak in non-tumoral cells; this is a dose-dependent effect, the data are statistically different at 75 µM. The distinct sub-G1 peak might represent condensed or fragmented DNA, which was stained with low level DNA fluorescence and further suggests an apoptotic death [5,6]. These data are in marked contrast to the effect of the peptide observed in melanoma and epidermoid carcinoma cells, in which no sub-G1 peak is evident. The analyses of the viability data after performing PI uptake assays (Fig. 3) showed that HaCaT cells were more sensitive to LTX-315 treatment than the cancer cells.

## 3 Experimental design, materials, and methods

### 3.1 LTX

LTX-315 (K–K–W–W–K–K–W–Dip–K–NH_2_, Lot.91799130007/PE4961) was synthesized by the solid-phase method according to the sequence published by Camilio et al. [4] and purchased from GenScript (Piscataway Township, NJ, USA). The purity of the peptide was determined by analytical HPLC to be higher than 95%, and the molecular weight was confirmed with MALDI-TOF mass spectrometry.

### 3.2 Cell culture

Human skin cancer cell lines, A375 and A431, were purchased from American Type Cell Culture (ATCC). The human keratinocyte line HaCaT was employed as normal cell control. Cells were cultured at a final concentration of 2.5 × 10^5^ cells/ml in Dulbecco's modified Eagle's medium (DMEM, Gibco). The medium was complemented with 5% fetal bovine serum (FCS, Gibco) and 1% penicillin/streptomycin (Gibco). Cell cultures were incubated at 37 °C with 5% CO_2_.

### 3.3 Treatment outline

Cells were disaggregated using trypsin-EDTA. Cell suspensions were centrifuged at 3500 rpm for 5 min, the supernatant was discarded and cell pellets were plated in 6-well plates at a final concentration of 2.5 × 10^5^ cells/ml. After adhesion, cells were treated with different concentrations of LTX-315 (25–75 µM) for 24 h and were then processed for cell cycle and cell viability. Peptide concentrations were selected based on previous studies of LTX-315 on different tumoral cell lines [3,[7], [8], [9], [10]. In these studies, concentrations ranging from 8 to 400 µM were evaluated. However, a maximum concentration of 75 µM was considered appropriate to induce changes in the cell cycle.

### 3.4 Cell cycle analyses

Measuring the DNA content of cells is a well-established method to monitor cell cycle [11]. In order to analyze cell progression through the cycle, exponentially growing cells were exposed to 25 , 50  or 75 µM of LTX-315 for 24 h, washed with PBS, and then disaggregated using trypsin-EDTA. One million cells per milliliter were suspended in ice-cold 70% ethanol and fixed for 1 h at 4 °C. Next, cell suspensions were washed in PBS, treated with 100 µg/ml RNase, and incubated with 100 µg/ml propidium iodide (PI) for 30 min. PI fluorescence of 10,000 events was analyzed using a BD LSRFortessa flow cytometer. Percentages of cells in each phase were calculated using FlowJo. Each experiment was performed three times.

### 3.5 PI staining (cell viability analysis)

One of the best markers for cell death is membrane integrity. In flow cytometry, plasma membrane permeability is assessed using propidium iodide, a dye that is not permeant to live cells [12]. To assess cell viability after 24 h of 25 , 50  or 75 µM LTX-315 treatments, cells were washed with PBS and disaggregated using trypsin-EDTA. Cell pellets were resuspended, stained with 1 mg/ml PI, and incubated in the dark at room temperature for 20 min. Following this, 10,000 events were analyzed by flow cytometry in BD LSRFortessa. Fluorescence was quantified using FlowJo. Each experiment was performed three times.

### 3.6 Statistical analysis

Data were expressed as mean ± standard error of the mean. ANOVA analyses were performed using via GraphPad Prism, with post-hoc comparisons carried out by Fisher's protected least significant difference (FPLSD) tests. *p* ≤ 0.05 was considered statistically significant.

## Appendix Supplementary materials

Image, application 1
